# RetSynth: determining all optimal and sub-optimal synthetic pathways that facilitate synthesis of target compounds in chassis organisms

**DOI:** 10.1186/s12859-019-3025-9

**Published:** 2019-09-09

**Authors:** Leanne S. Whitmore, Bernard Nguyen, Ali Pinar, Anthe George, Corey M. Hudson

**Affiliations:** Sandia National Laboratories, East Avenue, Livermore, 94550 USA

**Keywords:** Mixed integer linear programming, Metabolic engineering, Flux balance analysis

## Abstract

**Background:**

The efficient biological production of industrially and economically important compounds is a challenging problem. Brute-force determination of the optimal pathways to efficient production of a target chemical in a chassis organism is computationally intractable. Many current methods provide a single solution to this problem, but fail to provide all optimal pathways, optional sub-optimal solutions or hybrid biological/non-biological solutions.

**Results:**

Here we present RetSynth, software with a novel algorithm for determining all optimal biological pathways given a starting biological chassis and target chemical. By dynamically selecting constraints, the number of potential pathways scales by the number of fully independent pathways and not by the number of overall reactions or size of the metabolic network. This feature allows all optimal pathways to be determined for a large number of chemicals and for a large corpus of potential chassis organisms. Additionally, this software contains other features including the ability to collect data from metabolic repositories, perform flux balance analysis, and to view optimal pathways identified by our algorithm using a built-in visualization module. This software also identifies sub-optimal pathways and allows incorporation of non-biological chemical reactions, which may be performed after metabolic production of precursor molecules.

**Conclusions:**

The novel algorithm designed for RetSynth streamlines an arduous and complex process in metabolic engineering. Our stand-alone software allows the identification of candidate optimal and additional sub-optimal pathways, and provides the user with necessary ranking criteria such as target yield to decide which route to select for target production. Furthermore, the ability to incorporate non-biological reactions into the final steps allows determination of pathways to production for targets that cannot be solely produced biologically. With this comprehensive suite of features RetSynth exceeds any open-source software or webservice currently available for identifying optimal pathways for target production.

**Electronic supplementary material:**

The online version of this article (10.1186/s12859-019-3025-9) contains supplementary material, which is available to authorized users.

## Background

The biological production of compounds for industrial applications is an interesting and complex problem. From the perspective of biological retrosynthesis, there are essentially two challenges 1) identifying new enzymes to perform difficult and/or important chemical reactions and 2) determining the optimal (minimal) number of gene additions that is required to convert an industrial organism into one capable of successfully producing a compound of interest. There is a growing body of literature for solving the first problem and recent work on polyketide design has demonstrated considerable success [1]. This paper is focused on the second problem, which we argue is essentially a routing challenge. Identifying the minimal number of gene additions (herein referred to as an optimal pathway) has cost and time saving benefits in downstream production. Producing a compound of interest (hereafter *x*), not native in an organism requires determining the reaction (and corresponding enzyme/genes) additions necessary to produce *x*. Without complex routing algorithms the number of possible optimal pathways grows exponentially relative to the pathway length. As new biological reactions enter the literature and are available for synthetic addition, the optimal pathways may fork down completely different routes. Furthermore, there may be scenarios where the yield of a given compound is optimized, but the number of gene additions are sub-optimal (pathways with a greater number of gene/enzyme additions than the minimal). These all represent the distinct challenges in determination of pathways to production.

Reaction additions and subsequent optimal pathways can inefficiently be determined computationally by one-by-one addition of non-native reactions to a stoichiometric matrix for a chassis organism, and then performing flux balance analysis (FBA) to determine if there is compound production without interfering biomass production. FBA is a tool widely used in predicting genome-scale metabolic behavior [2]. FBA is principally used for its ease of setup and efficient optimal search. At a minimum, FBA requires a stoichiometric matrix (S) which is complete with regard to the available reactions and compounds for a given organism. The reactions are conventionally tied to a set of explicit enzymes and transporters. FBA uses linear programming, requiring an objective function (Z), to solve for the metabolism of interest. This may involve minimization of input, maximization of output, or other constraints [3].

Given *k* reactions to produce *x*, the naive approach to adding new reactions is to search each of the *k* reactions in the database to see if *x* is produced given the available compounds from FBA. This requires query of each of the *k* reactions. If there is a single step solution, it solves in FBA(*k*) time. Where there are no single step solutions, the problem explodes exponentially. A two-step solution requires not just *k* reactions, but all reactions that produce precursors to the *k* reactions. If the average number of reactions producing a given compound is $\overline {g}$, the number of pathways that must be tested for a *y* step solution in the worst case is $\text {FBA}(\overline {g}^{y})$.

RetSynth overcomes the naive and inefficient method of identifying solutions, particularly the worst-case, using constraint based mixed-integer linear programming (MILP). Given a database of known biological and chemical reactions and a genome-scale metabolic model, which can be constructed using RetSynth from numerous metabolic repositories with known enzymatic and chemical transformations, all optimal genetic additions required to produce a given compound of interest can be determined. The manner in which MILP is implemented is to minimize the objective value which represents the number of steps in the pathway. While selecting pathways based on number of reaction steps does not account for other issues in synthetic pathways (such as enzyme efficiency, enzyme or compound toxicity, or target yield) this is an ideal starting method for identifying synthetic pathways as minimizing the alterations made to a chassis organism is likely to lessen the above-mentioned issues as well as be more cost effective. Additionally, by resetting weights for reactions in the optimal pathway, RetSynth will automatically find novel sub-optimal pathways thereby providing alternative pathways that may have better target yield or fewer toxicity problems. This can be performed iteratively to determine all sub-optimal pathways for a specific path length.

Herein we describe the algorithm developed as part of RetSynth to efficiently provide solutions targeted compound production. Subsequently, RetSynth can determine which pathway will produce the highest yields of a target compound using FBA. With this comprehensive suite of features, RetSynth is an efficient tool for identifying optimal solutions to target compound synthesis. Additionally, we compare RetSynth performance to other tools that can find optimal pathways to target compound production, such as OptStrain [4], MetaRoute [5], GEM-Path [6], ReBIT [7], RetroPath [8], and RouteSearch [9]. RetSynth outperformed these tools in overall capabilities including, identifying more optimal and sub-optimal pathways, evaluating pathway efficiencies using FBA, the number of metabolic repositories it can compile into a single concise metabolic database, and the time necessary to identify optimal and sub-optimal pathways. Identification of sub-optimal pathways allows the user more pathway choices than other algorithms currently provide, while not producing an overwhelming number of solutions. The ability to provide optimal and sub-optimal solutions is unique to RetSynth and to our knowledge does not currently exist in other available tools.

## Implementation

RetSynth includes a comprehensive suite of features necessary for complete implementation of the software. To find pathways RetSynth requires a metabolic database of reaction (i.e. corresponding catalytic gene/enzyme information) and compound information. RetSynth can construct a database of metabolic information from number of metabolic repositories, including PATRIC [10, 11], KBase [12], MetaCyc [13], KEGG (Kyoto Encyclopedia of Genomes and Genes) [14], MINE (Metabolic In-Silico Network Expansion database) [15], ATLAS of Biochemistry [16] and SPRESI [17]. Additionally, users can add individual reactions to the database. These may be newly discovered from the literature or proprietary reactions. Combining biological and chemical reaction repositories into one database allows RetSynth to construct a comprehensive and concise metabolic database. In order to rank discovered pathways based on target yield in a chassis organism, RetSynth uses CobraPy [18] to perform FBA. The results are conveniently rendered with a visualization module, allowing the user to quickly interpret results. RetSynth is a stand-alone software package, built with Pyinstaller, which does not require a webservice or MATLAB, entirely written in Python except for two required non-Python dependencies, the GNU Linear Programming Kit (http://www.gnu.org/software/glpk), and libSMBL [19]. Finally, we have built an easy-to-use graphical user interface to make RetSynth usable by everyone.

## Results

### RetSynth algorithm

The algorithm described below was developed for the RetSynth software to rapidly and efficiently identify all optimal pathways to target compound production in a specified chassis organism. Optimal pathways can then be ranked based on their ability to produce the highest yields of a compound by evaluating flux through each candidate pathway.

To identify optimal pathways, we constructed a MILP:

1 $$\begin{array}{*{20}l} & \text{minimize} \qquad z=\mathbf{t}^{\mathrm{T}} \mathbf{x}\\ & \text{s.t.} \qquad \qquad \,\,\mathbf{Cx = d},  \\ & \text{and} \qquad \qquad \mathbf{x} \in \text{\{0,1\}}^{m},  \end{array} $$

where the entire RetSynth metabolic database is represented by a stoichiometric matrix C, with dimensions *m* molecules ×*n* reactions which are in the database. *x* is a vector of variables the length of *n* which represent the presence or absence (1 or 0) of each reaction in an optimal path. *Cx*=*d* where *d* is a vector of the length *m* which sets bounds on metabolite availability depending on whether the molecule is a native metabolite to the chassis organism (*n*) which is not constrained, a non-native metabolite (*w*) which constrains the molecule to ensure if the molecule is consumed in the optimal path it has to also be produced by a reaction in the optimal path or the target molecule (*g*) which has to be produced by a variable (2).

2 $$ \begin{aligned} n = \left[\begin{array}{l} \infty \\ \infty \\ \vdots \\ \infty\\ \end{array}\right] w = \left[\begin{array}{l} \geq 0 \\ \geq 0 \\ \vdots \\ \geq 0\\ \end{array}\right] g = \left[\begin{array}{l} 1 \\ \end{array}\right] d = \left[\begin{array}{l} n \\ w \\ g \\ \end{array}\right] \end{aligned}  $$

The objective function is set to minimize the number of variables (reactions) needed to produce the target compound. The objective function weights are distributed based on whether the variables (reactions) are native (*I*, vector of weights for native variables) or not native (*E*, vector of weights for non-native variables) (3).

3 $$ \begin{aligned} I = \left[\begin{array}{l} 0 \\ 0 \\ \vdots \\ 0\\ \end{array}\right] E = \left[\begin{array}{l} 1 \\ 1 \\ \vdots \\ 1\\ \end{array}\right] t = \left[\begin{array}{l} I \\ E \\ \end{array}\right] \end{aligned}  $$

To identify all the optimal pathways, a penalty function is added to variables that are already identified as part of an optimal pathway, forcing the algorithm to seek an alternative optimal pathway. To implement this algorithm, *S*^*v*^ is the total set of variables and $S^{*}_{v}$ is a subset of variables in an optimal pathway. We compute the penalty such that any optimal pathway to the modified problem remains an optimal pathway to the original problem, that is *t*^*T*^*x*<*β*^∗^(1+1/(2*β*^∗^)<*β*^∗^+1, where *β*^∗^ is the number of reaction steps in the optimal pathway.

Here we illustrate how variables are weighted given that they are in an identified optimal pathway $S^{*}_{v}$. Assume the *j*th variable is a part of an optimal pathway but is not included in $S^{*}_{v}$. Then we have *t*_*j*_=1. The weights in *t* for the other *β*^∗^−1 variables that are part of the optimal pathway are 1+1/(2*β*^∗^). All together the optimal pathway value to the modified problem will be *β*^∗^+1/2−1/(2*β*^∗^). The algorithm terminates only after the objective function value to the modified problem reaches *β*^∗^(1+1/(2*β*^∗^)), which is higher than the pathway that includes the *j*th variable (Algorithm 1). This leads to a contradiction and proves that our algorithm includes all variables that are part of an optimal pathway. 
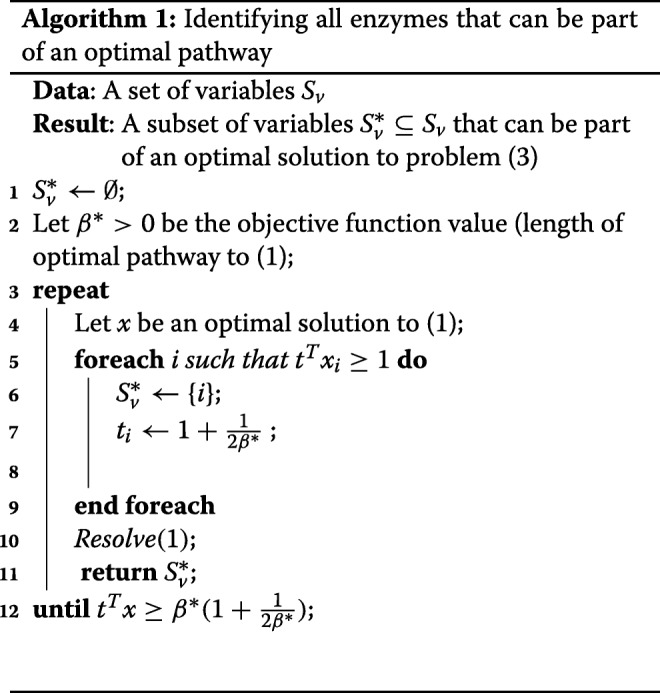


#### Sub-optimal length pathway enumeration

RetSynth is able to find pathways that are not only optimal, but pathways up to *β*^∗^+*k*, where *k* is a parameter set by the user and indicates the level of sub-optimal pathways to be identified. This involves adding additional constraints to (1) which prevents any of the initial optimal pathways from being discovered, forcing the algorithm to seek the next best pathway. For each initial optimal pathway, a constraint is added:

4 $$ \begin{aligned} Y = \left[\begin{array}{l} 0 \\ 0 \\ \vdots \\ 0\\ \end{array}\right] O = \left[\begin{array}{l} 1 \\ 1 \\ \vdots \\ 1\\ \end{array}\right] P = \left[\begin{array}{l} Y \\ O \\ \end{array}\right] \end{aligned}  $$

where *Y* are variables that are not part of a given optimal pathway and *O* are variables in an optimal pathway $S^{*}_{v}$. Combining vectors *Y* and *O* results in vector *P* (4). Constraints are set so that the combination of reactions in the optimal pathway cannot be identified as a solution. With the new constraints the metabolic system is:

5 $$\begin{array}{*{20}l} & \text{minimize} \qquad z=\mathbf{t}^{\mathrm{T}} \mathbf{x} \\ & s.t. \qquad \mathbf{Cx = d},  \\ & \qquad \qquad {foreach}\ \beta^{*}\ \text{in optimal solutions:}  \\ & \qquad \qquad \qquad \mathbf{P}^{\mathrm{T}} \mathbf{x} \leq \beta^{*}-1  \\ & \text{and} \qquad \mathbf{x} \in \text{\{0,1\}}^{m}  \end{array} $$

Adding these constraints forces the algorithm to seek the next best sub-optimal pathway (5). At each level, *k* constraints are added to prevent the algorithm from finding previous levels of optimal or sub-optimal pathways. For each level of *k* algorithm (1) is implemented to identify all sub-optimal pathways at that level, with the exception that instead of resolving algorithm (1) it is resolving (5).

After all optimal and sub-optimal solutions are identified, pathways are integrated into an FBA model for the chassis organism and FBA is run optimizing growth (the biomass reaction) and production of the target compound [2, 18].

#### Enumerating and backtracking all solutions

The new set $S_{v}^{*}$ is typically much smaller than *S*_*v*_, and drastically reduces the search space for enumerating all optimal solutions. To track optimal paths, define a directed graph *G*=(*V*,*E*) with two types of nodes: *V*=*V*_*c*_∪*V*_*p*_ and *V*_*c*_∩*V*_*p*_=*∅*. The process nodes *V*_*p*_ represent the enzymes selected in the previous section, whereas the compound nodes *V*_*c*_ represent all compounds that are inputs to the processes. Directed edges represent the input/output relationships between compounds and processes. The backtracking proceeds by starting with target compound *x*. Step 1 is to determine processes in *V*_*p*_ that produce *x*. A directed edge is connected between nodes in *V*_*p*_ and *x*. These nodes are then removed from *V*_*p*_. Step 2 is to determine compounds that serve as inputs for these removed nodes and to add them from *V*_*c*_. If *V*_*p*_ is not empty, step 1 will be repeated for each added node from *V*_*c*_. This process will be repeated until *V*_*p*_ is empty, resulting in a directed dependency graph *G* of all pathways to production by native metabolism to *x*.

*Given a compound of interest and a dependency graph G, a connected subgraph that includes the node for the compound of interest and at least one predecessor node for each compound node describes a feasible solution to the problem. Symmetrically, any feasible solution is a subgraph that satisfies these conditions. Subsequently, such a subgraph with minimum number of process nodes defines an optimal solution.*

### Validating RetSynth

Using metabolic networks from KBase and data from the MetaCyc metabolic repository, RetSynth was used to identify optimal pathways for compounds which already have experimentally tested synthetic pathways in *Escherichia coli*. Comparing model results to experimentally validated pathways demonstrates that RetSynth can generate practical candidate pathways for compound synthesis.

**Fig. 1:**
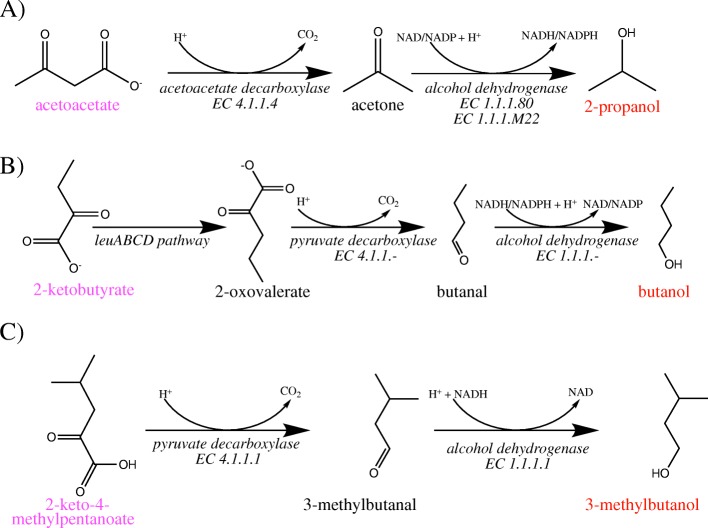
RetSynth Validation. Optimal pathways identified by RetSynth for 2-propanol (**a**), butanol (**b**) and 3-methylbutanol (**c**). Red indicates compound targets, magenta indicates native compounds to *Escherichia coli* K-12 M1655 or BW25113

2-propanol has previously been produced in *Escherichia coli* JM109 grown on LB media. Enzymes were added into *E. coli* in order to convert the native precursor acetyl-CoA into 2-propanol [20]. These conversions include acetyl-CoA to acetoacetyl-CoA, acetoacetyl-CoA to acetoacetate, acetoacetate to acetone, and finally acetone to 2-propanol. Enzymes thiolase, CoA-transferase, acetoacetate decarboxylase and alcohol dehydrogenase were added to *Escherichia coli* JM109 to facilitate these reactions. For RetSynth, the chassis organism *Escherichia coli* strain K-12 M1655 was used because a metabolic model for strain JM109 was not freely available. The optimal pathway identified by RetSynth consisted of the catalytic conversions acetoacetate to acetone and acetone to 2-propanol (acetoacetate decarboxylase and alcohol dehydrogenase catalyzed these reactions, respectively) (Fig. 1A). Though shorter because the *Escherichia coli* K-12 M1655 strain has acetoacetate (which needs to be synthetically produced in *Escherichia coli* JM109) RetSynth’s optimal pathway uses the overall production pathway shown by Jojima et al. to be effective in producing 2-propanol [20].

To produce 1-butanol in *Escherichia coli* BW25113 on an M9 media, Atsumi et al. added a synthetic pathway consisting of 3 enzymatic conversions starting with the conversion 2-ketobutyrate to 2-oxovalerate [21]. Because 2-ketobutyrate is a rare metabolite in *Escherichia coli* BW25113, the authors add an overexpressed leuABCD pathway to increase yields of this precursor. Subsequently, 2-oxovalerate is converted to butanal by pyruvate decarboxylase and then to butanol by alcohol dehydrogenase. Using the standard BW25113 metabolic model retrieved from the KBase repository, RetSynth was unable to identify this pathway since the model did not contain a reaction for 2-oxovalerate synthesis. The lack of production of this metabolite in the model is unsurprising as natural yield of the precursor is so minimal in *Escherichia coli* [21]. However, with the capabilities of RetSynth, it is simple to manually add this pathway into the model, as Atsumi et al. did to increase production of 2-oxovalerate. Once the leuABCD pathway was added, the same pathway was identified by RetSynth as was published by Atsumi et. al (Fig. 1b).

Our third validation example was to find the optimal pathway to production of 3-methylbutanol in *Escherichia coli* strain BW25113. Our pathway converted native metabolite 2-keto-4-methylpentanoate to 3-methylbutanal and then subsequently produced 3-methylbutanol via added enzymes pyruvate decarboxylase and alcohol dehydrogenase (Fig. 1C). This matches the synthetic pathway used by [20] to produce 3-methylbutanol.

### Optimal and sub-optimal pathways for MetaCyc compounds in *Escherichia coli* K-12 M1655

The power of RetSynth lies in its ability to quickly identify optimal and sub-optimal pathways for a large set of target compounds. To illustrate this strength, a database was constructed consisting of a KBase metabolic network for *Escherichia coli* K-12 M1655 and MetaCyc reaction information. For every compound in the MetaCyc repository that was not native to *Escherichia coli* K-12 M1655, RetSynth identified an optimal pathway along with two levels (pathways that require more than the minimal number of gene additions, specifically, second and third best number of gene/reaction additions) of sub-optimal pathways.

**Fig. 2:**
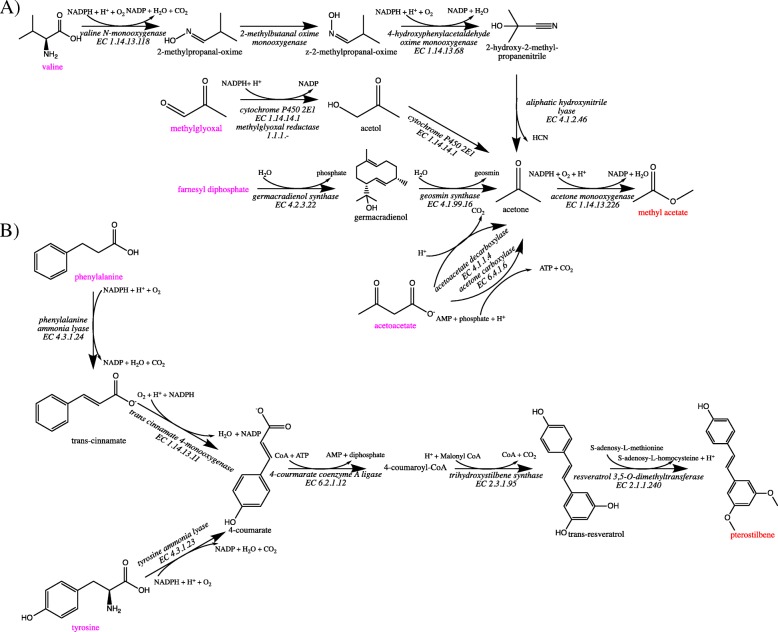
Optimal and sub-optimal pathways. Optimal and sub-optimal pathways identified by RetSynth for methyl acetate (**a**), and pterostilbene (**b**). Red indicates compound targets, magenta indicates native compounds to *Escherichia coli* K-12 M1655

Of the 15,706 MetaCyc compounds that were not native to *Escherichia coli* K-12 M1655, we found synthetic pathways for 3462 compounds. Optimal and sub-optimal pathways for methyl acetate and pterostilbene, both of which have economic value, are illustrated in Fig. 2. For methyl acetate, which is commonly used in paints and nail polish, optimal and two levels of sub-optimal pathways were identified for production in *Escherichia coli*. The optimal pathway synthesizes acetone from the native compound acetoacetate and subsequently converts acetone to methyl acetate (Fig. 2a). The last step of the optimal pathway is then shared among all candidate pathways. The two-level sub-optimal pathways include the conversion of the native compound farnesyl diphosphate to acetone and the conversion of methylglyoxal to acetone through two enzymatic steps. The level two sub-optimal pathway synthesizes 2-methylpropanal-oxime from the native compound valine which is then followed by three enzymatic conversions to produce acetone. The second target compound pterostilbene, which has been shown to have health benefits such as lowering cholesterol and glucose levels [22], can be synthesized in *Escherichia coli* through the identified optimal pathway, which consists of four enzymatic conversions starting with the native compound tyrosine, or the level one sub-optimal pathway, which has five enzymatic conversions starting with phenylalanine (Fig. 2b). A second level sub-optimal pathway could not be identified for this compound. Theoretical yields were predicted using RetSynth’s FBA module to be 0.24 and 0.02 (mol/mol of glucose) for methyl acetate and pterostilbene, respectively. These compounds are just two examples of the 3462 compounds that we were able to quickly and efficiently discover optimal and sub-optimal pathways.

**Fig. 3:**
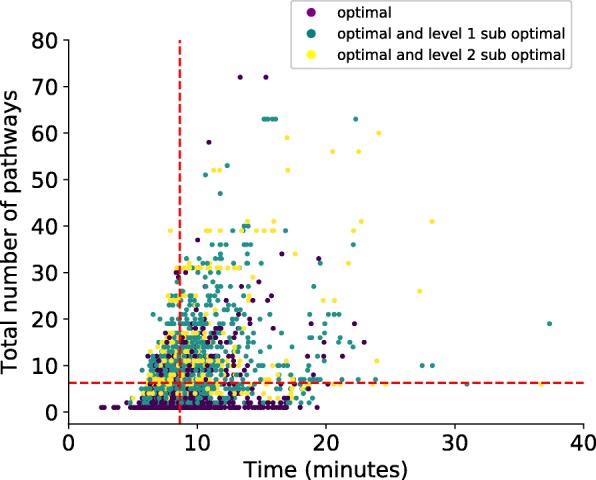
Optimal and sub-optimal pathways. Number of pathways versus time for each target compound. Red dashed lines indicate the averages on the Y and X axis. Colors indicate whether optimal and sub-optimal (level 1 and 2) pathways (yellow), optimal and sub-optimal (level 1) pathways (teal) or optimal pathways only (purple) could be identified for each compound

Of the 3462 targets, 513 compounds had optimal and sub-optimal level one and two pathways, 1125 compounds had optimal and sub-optimal level one pathways, and for the remaining 1824 compounds only had optimal pathways. The average number of pathways identified for a compound was 7 and the average time it took to calculate all pathways for a compound was 8 minutes (Fig. 3). Some compounds significantly exceeded the average time, which is due to the process of eliminating cyclic pathways. When a cyclic pathway is identified, constraints must be added to the MILP to prevent the pathway from being identified as a viable route to production (Additional file 1). The MILP is then resolved to calculate an alternative pathway. Thus, compounds with multiple cyclic pathways dramatically increase the time required to find optimal routes to production.

**Fig. 4:**
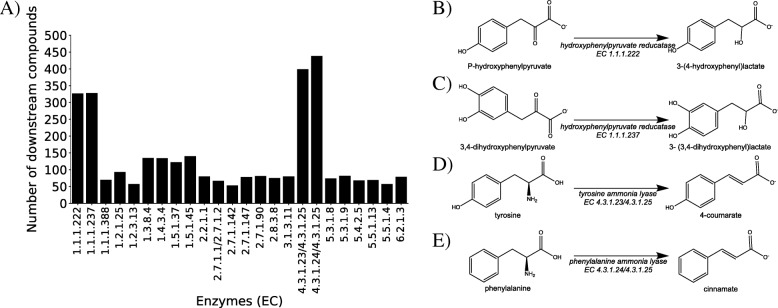
Optimal enzyme/gene addition. **a** Depicts the number compounds each enzyme is in an optimal or sub-optimal pathway (only shows enzymes that are in 50 or more compound pathways). **b**, **c**, **d**, **e** Are the reactions that are catalyzed by the top four enzymes in the highest number of compound pathways

Using the RetSynth results for the 3462 target compounds, we can identify which reaction/enzyme is common to the highest number of them. This gene would be an advantageous gene addition for cultured strains of *Escherichia coli*. To identify what reaction/enzyme would make an optimal genetic modification (i.e. leading to the production of the highest number of downstream targets, given that subsequent genetic modifications were made) for each reaction/enzyme we counted the number of compounds for which it was the first step in an optimal or sub-optimal pathway. Each reaction/enzyme was only counted once per compound even if it was in multiple optimal and/or sub-optimal pathways. Of the total 766 enzymes that were the first step in optimal and/or sub-optimal pathways, we identified 24 enzymes that were in 50 or more compound production pathways (Fig. 4a). The top four reactions/enzymes found in the highest number of target compound pathways, above 100 compounds, are illustrated in (Fig. 4b, c, d, e). Enzymes 1.1.1.222 and 1.1.1.237 are hydroxyphenylpyruvate reductases which catalyze the reactions in Fig. 4b and c respectively and are natively found in *Solenostemon scutellarioides*. The remaining two enzymes 4.3.1.23 and 4.3.1.24 (tyrosine ammonia-lyase and phenylalanine ammonia-lyase respectively) catalyze reactions in Fig. 4d and e. These enzymes are natively found in organisms *Rhodotorula glutinis* and *Ustilago maydis* respectively. Additionally, it was discovered that enzyme 4.3.1.25 can catalyze both these reactions and is found in *Rhodotorula glutinis*. By identifying enzyme additions that are in the highest number of target compound production pathways RetSynth can lead and enhance the development of efficient chassis organisms for optimal production of all types of economically and industrial target compounds.

### Biological and chemical hybrid pathways for target compound production

**Fig. 5:**
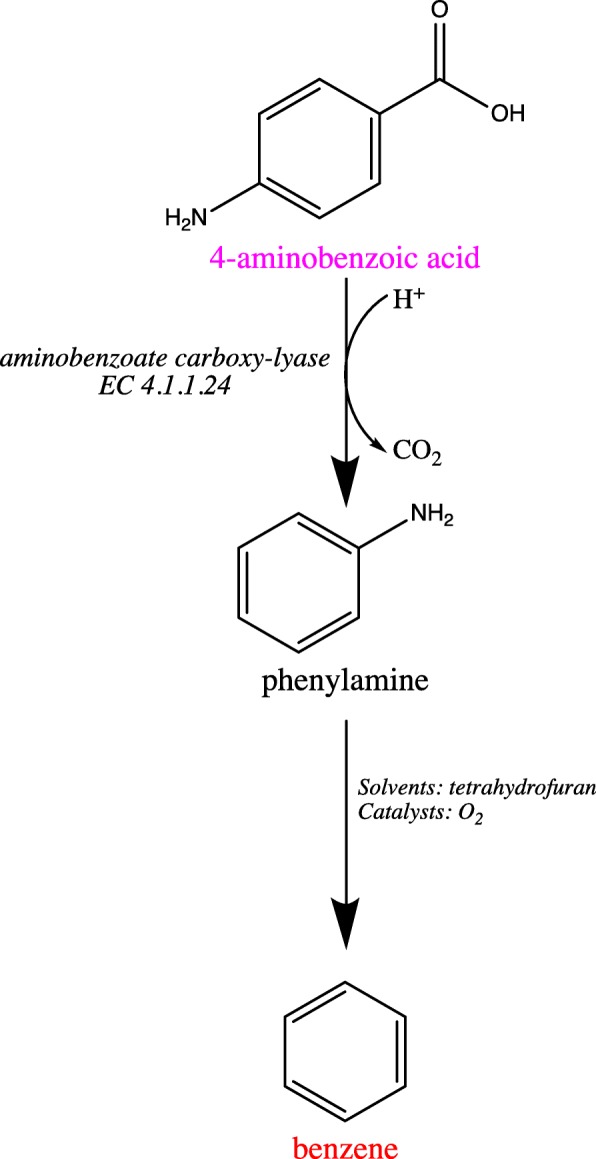
Optimal pathway for benzene production. Hybrid pathway including biological and chemical reactions necessary to produce benzene. Red indicates compound targets, magenta indicates native compounds to *Escherichia coli* K-12 M1655

In addition to identifying biological optimal and sub-optimal pathways, RetSynth can incorporate strictly synthetic chemistry reaction repositories such as SPRESI, which contains thousands of chemical reactions, into its metabolic database. By integrating SPRESI into RetSynth’s MetaCyc and KBase database, pathways that use both biological and chemical reactions to produce necessary compounds (termed hybrid pathways) can be discovered. With the addition of SPRESI, 413 more target compound production pathways were identified. The hybrid pathway for production of benzene in *Escherichia coli* K-12 M1655 (Fig. 5) consists of the enzymatic conversion of native compound 4-aminobenzoic acid to phenylamine (predicted theoretical yield to be 0.24 mol/mol glucose) which can subsequently be chemically synthesized into benzene [23]. Benzene is an important precursor to the production of other high value compounds. The ability to build a hybrid database greatly expands RetSynth’s capability for the find pathways to production of many target compounds that would otherwise not be possible.

## Discussion

### Benchmarking RetSynth to other pathway identifying tools

There are a number of other tools which can find synthetic pathways for target compounds, however none of these tools encompass all of the features of RetSynth (Table 1). We perform comparisons between RetSynth and other tools to illustrate RetSynth’s increased number and improved capabilities by benchmarking features between software such as the number of pathways found for each target compound, predicting yield of each target (if applicable) and time required to obtain results.

**Table 1:** Comparison of different software

Software	Easily available	Optimal pathways	Sub-optimal pathways	Chemical optimal/suboptimal pathways	Flux balance analysis	Graphical user interface	Enzyme promiscuity
RetSynth	*√*	*√*	*√*	*√*	*√*	*√*	
OptStrain		*√*	*√*		*√*		
GEM-Path		*√*					*√*
MetaRoute	*√*	*√*				*√*	
RouteSearch	*√*	*√*	*√*			*√*	
Retrobiosynthesis		*√*					
RetroPath	*√*	*√*	*√*			*√*	*√*

Depicts the different features of each software

#### OptStrain

OptStrain uses mixed integer linear programming (optimization-based framework) to find stoichiometrically balanced pathways that produce a target compound in a specified chassis organism [4]. The design flow for this software follows three main steps: 1) generation of a metabolic database filled with stoichiometrically balanced reactions from four metabolic repositories (KEGG, EMP (Enzyme and Metabolic Pathways), MetaCyc, UM-BBD (University of Minnesota Biocatalyst/Biodegradation database), 2) calculation of the maximum theoretical yield of the target compound with no restriction on whether native or non-native reactions are used, and 3) identification of the pathway that minimizes the number of non-native reactions and maximizes theoretical yield. Additionally, OptStrain identifies alternative pathways that meet both the criteria of minimization of non-native reactions and maximum theoretical yield. Because the software is no longer supported, a direct comparison to RetSynth could not be performed. However, there are numerous key differences between the two software. RetSynth allows the user direct control of the pathways they identify, specifically the level of sub-optimal pathways to find, and does not directly tie them to the yield of the target compound which ultimately results in a more comprehensive list of synthetic pathways to evaluate. The user also has more ability to add a variety of different types of reactions and compounds to the RetSynth database, including those from the literature that are not yet in a repository, as well as chemical reactions. Integrating chemical reactions into the database permits the user to also identify hybrid (containing both biological and chemical reactions) pathways. Because all targets cannot be produced biologically, this gives the user more pathways than would have otherwise be achieved using OptStrain. Additionally, the overall usability of RetSynth far surpasses OptStrain’s, primarily because RetSynth has an easy-to-use graphical user interface and is a stand-alone software package, precluding the need for any knowledge of programming or command-line usage. Overall, these features of RetSynth result in a more comprehensive and functional tool than what OptStrain currently provides.

#### GEM-Path

The GEM-Path algorithm uses several different techniques to design pathways for target compound production in a chassis organism [6]. This algorithm specifically uses 443 reactions that were pulled from BRENDA and KEGG repositories to identify pathways in *Escherichia coli*. The 443 reaction were methodically classified into three different categories 1) reactions that use no co-substrates or co-factors, 2) reactions that are anabolic conversions (merging the substrate with a co-substrate), and 3) reactions that are catabolic conversions where the substrate breaks down into corresponding product and co-product. Additionally, thermodynamic analysis was performed for each reaction, calculating *ΔG* (KJ/MOL), as was a promiscuity analysis (determining if an enzyme could accept multiple substrates). Subsequently, GEM-Path implemented a pathway predictor algorithm, which works by 1) designating a target compound and setting predictor constraints (maximal pathway length, metabolites to compute at each iteration, thermodynamic threshold, and reaction promiscuity threshold), 2) applying reactions to the target in a retrosynthetic manner for generating the corresponding substrates, and 3) checking if the substrate matches a compound in the *Escherichia coli* metabolome. Subsequently, if a pathway is found FBA is run to validate production.

GEM-Path is not available for public use and there are other differences between the two software. GEM-Path integrates more detailed reaction parameters when predicting a pathway (i.e. *ΔG* and promiscuity) than RetSynth uses to identify optimal solutions. This subsequently makes GEM-Path’s metabolic database substantially smaller than RetSynth and therefore is missing many synthetic pathway opportunities. Additionally, GEM-Path’s algorithm does not allow multiple pathways per target to be identified, limiting the potential pathways provided to the researcher.

#### MetaRoute

MetaRoute is a web-based tool that finds pathways between two specified compounds using a graph-based searching algorithm [5]. Specifically, this tool uses Eppstein’s k-shortest path algorithm to find the shortest distance between two nodes in a graph. The graph representing a metabolic network was built by 1) using pre-calculated and concise atom mapping rules in which two successive reactions are represented by a single edge, 2) removing irrelevant reaction conversions (i.e. glucose 6 phosphate to ATP to AMP), and 3) using an updated weighting schema which decreased weights on edges through frequently used metabolites which traditionally had higher weights. The graph of reactions and compounds MetaRoute uses was built using several metabolic repositories including BN++ (a biological information system), BNDB (biochemical network database) and KEGG. There are several key differences between this web-based tool and RetSynth, one being that a source compound must be specified instead of a chassis organism, which limits the number of pathways that can be discovered. While a user could perform a pathway search between every internal chassis compound and the target, this would take an extraordinary amount of time to get all optimal pathways and require the user to further sort through the pathways and identify the best route. Additionally, this is not a tool that can find sub-optimal pathways or evaluate the effectiveness of pathways through FBA. RetSynth’s capabilities far exceed MetaRoute’s including being a stand-alone software package that does not require a webservice like MetaRoute.

#### RouteSearch

RouteSearch is a module of the Pathway Tools software utilizing the EcoCyc and MetaCyc databases for synthetic pathway identification [9]. This tool uses the branch-and-bound search algorithm on atom mapping rules to find optimal pathways between a set of starting compounds (or a specified source compound) and a target compound. Users can specify the weights (costs) of identifying pathways with reactions native to the chassis organism and those external to the organism. Additionally, multiple optimal pathways as well as higher cost or length sub-optimal pathways can be identified by RouteSearch. The user must specify how many pathways they want to examine, and if there are fewer optimal pathways than the user specified, then RouteSearch will give longer (sub-optimal) pathways. When identifying pathways by RouteSearch using the BioCyc web-browser a set of source compounds can be used to find pathways to an individual target compound. Additionally, a number of external bacterial organisms can be set by the user in which to search for optimal pathways. When using all bacterial organisms, however, RouteSearch freezes and is unusable. In addition to the web browser, RouteSearch can be used through the Pathway Tools software suite, which allows all MetaCyc reactions to be loaded quickly and efficiently. When using RouteSearch through Pathway Tools only a single source compound can be set and optimal pathways cannot be identified from an entire set of source compounds. Thus a rapid search for an optimal and sub-optimal pathway using all native chassis organism metabolites cannot be rapidly or efficiently achieved. While RouteSearch can perform similar functions to RetSynth the usability and system-wide analysis that RetSynth provides cannot be matched.

#### Retrobiosynthesis

Retrobiosynthesis is a synthetic biology tool that can build novel synthetic pathways for compound production. This tool, which was developed by the Swiss Federal Institute of Technology [24], first implements a network generation algorithm that compiles a list of all theoretically possible enzymatic transformations. A pathway reconstruction algorithm, using either a graph-based search or optimization-based methods, then builds all possible pathways from a source compound to a target. After implementation of these algorithms, reduction steps are taken to decrease the amount of information which include: 1) sorting through the list of possible enzymatic transformations and comparing what is known vs novel using repositories such as KEGG, MetaCyc, and ChEBI, and 2) sifting through the pathways and selecting ones based on thermodynamic feasibility, number of enzymatic transformations in a pathway and maximum target yield.

Although the Retrobiosynthesis tool performs many of the same functions as RetSynth, and can predict novel enzymatic transformations, its ability to be used by independent researchers is limited. It requires setting up a collaboration with the Swiss Federal Institute of Technology and having them run the analysis. Retrobiosynthesis requires a designation of a source compound, making it likely that identifying all pathways to a target in a chassis organism would require a large amount of time, although we could not test this as we do not have access to the tool. RetSynth is a stand-alone software with a graphical user interface that researchers can download and use independently, making identifying pathways less reliant on the developers. Overall the software is quicker and easier to use for researchers to find optimal pathways.

#### RetroPath

RetroPath is a synthetic pathway finding tool used to identify pathways between a set of source compounds and a target compound [8]. RetroPath uses a database (database named RetroRules) of external metabolic reactions which was constructed using reaction information collected from BNICE, Simpheny, KEGG, Reactome, Rhea and MetaCyc. Reactions are represented by reaction SMARTS which facilitates the ability for potential novel enzymatic transformations to be predicted. Pathways between source and target compounds are calculated by identifying the shortest hyperpath in a larger weighted hypergraph (constructed using the database of external reactions) using the FindPath algorithm [25, 26].

**Fig. 6:**
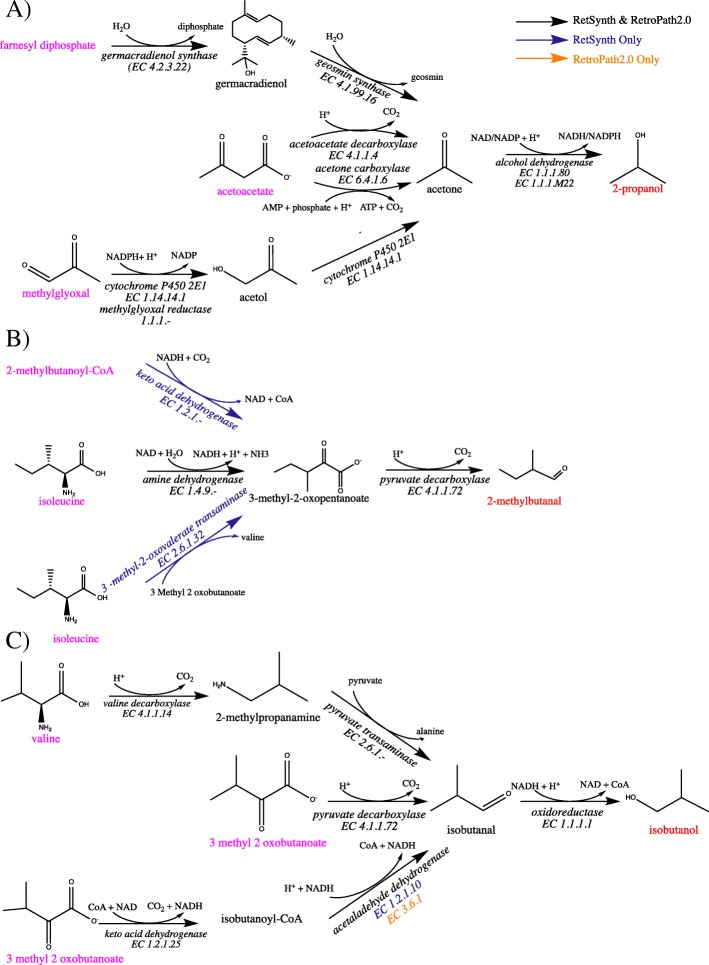
RetSynth vs RetroPath2.0. Optimal and sub-optimal pathways identified by RetSynth and RetroPath for 2-propanol (**a**), 2-methylbutanal (**b**) and isobutanol (**c**). Red indicates compound targets, magenta indicates native compounds to *Escherichia coli* K-12 M1655

To compare synthetic pathways between RetSynth and RetroPath we first retrieved the reaction SMARTS available for the MetaCyc repository from the RetroRules full database (https://retrorules.org/). A RetSynth database was then built to match the reactions that were in the RetroPath MetaCyc reaction rules database so an equal comparison between the tools could be run. Extra RetroPath parameters such as maximum and minimum diameter and maximum molecular weight for source were all kept at their default values of 1000, 0 and 1000 respectively. Diameter is a measure of the depth and detail of the molecular reaction signatures (reaction SMARTS) used to identify pathways in RetroPath. The larger diameter the more detailed and strict the reaction SMARTS are and therefore are less able to predict novel reactions. Because RetSynth cannot predict novel reactions and we want to do a strict comparison between the two tools the maximum diameter of 1000 keeps the reaction SMARTS sufficiently strict to prevent novel reactions from being identified by RetroPath. Additionally, source compounds (metabolites native to *Escherichia coli* K-12 M1655) were also the same for the two tools. Using RetroPath, which was run with the KNIME analytics platform with the pathway limit being 10 reaction steps (which matched the default pathway limit of RetSynth) we attempted to identify pathways for all MetaCyc compounds not in *Escherichia coli*. This query, however, was too large for RetroPath to handle, and subsequently RetroPath was employed to find pathways for a smaller set of target compounds including methyl acetate, pterostilbene (Fig. 2), 2-propanol, butanol, sabinene, 2-methylbutanal and isobutanol. RetSynth with this smaller database was able to identify pathways for all compounds in this smaller set while RetroPath was only able to find optimal and sub-optimal pathways for 2-methylbutanal, isobutanol and 2-propanol (Fig. 6).

RetSynth and RetroPath were able to identify 3 pathways for production of 2-propanol in *Escherichia coli* (Fig. 6a). Pathways identified by the tools consisted of 1) the conversion of native compound farnesyl diphosphate to 2-propanol in 3 enzymatic conversions, 2) the conversion of native compound acetoacetate to 2-propanol in 2 enzymatic conversions, and 3) the conversion of methylglyoxal to 2-propanol in 3 enzymatic conversions. Both tools were also able to find synthetic pathways for 2-methylbutanal (Fig. 2b). RetSynth was able to find 3 pathways, all of which contained 2 enzymatic steps. All pathways produce the intermediate 3-methy-2-oxopentanoate (which is subsequently converted to 2-methylbutanal) from 3 different native compounds including 2-methylbutanoyl CoA, isoleucine and 3-methyl-2-oxobutanoate. RetroPath was only able to identify one pathway which was the conversions of isoleucine to 3-methyl-2-oxopentanoate and then to 2-methylbutanal. Finally, for isobutanol 3 pathways of almost identical enzymatic conversions were found by RetroPath and RetSynth (Fig. 6c). Both identified the 3-step pathway which takes valine and produces isobutanol as well as a 2-step pathway which takes 3-methyl-2 oxobutanoate and produces isobutanol. The final pathway of 3 enzymatic conversion steps starts again with native compound 3-methyl-2-oxobutanoate and transforms it into isobutanoyl-CoA and then into isobutanal and subsequently isobutanol. The second step is catalyzed by EC 1.2.1.10 in RetSynth and EC 3.6.1.- in RetroPath2.0. The removal of CoA from a substrate is represented by a general reaction in RetroPath and therefore the corresponding enzyme is less specific than what is given by RetSynth.

Overall RetSynth was able to identify pathways for a larger set of compounds than RetroPath. Additionally, RetSynth’s supplementary capabilities, including identifying theoretical yields for target compounds as well as incorporating chemical reactions into the database of external reactions makes it highly versatile for individual user needs. RetSynth can be easily run using the graphical user interface and can implement usage of multiple processors, enabling quick identification of synthetic pathways for large sets of target compounds. Currently, RetSynth can only generate pathways with reactions that are known enzymatic transformations while RetroPath, by having a database of reaction SMARTS allows the software to predict novel enzyme transformations. While this RetroPath feature undoubtedly has advantages in discovering production pathways, the goal of RetSynth is to provide the most feasible pathways for target production and therefore using known reactions ultimately makes pathways provided by RetSynth more likely to be functional. Furthermore, because RetSynth is a stand-alone software package it is extremely easy to use and does not require downloading any outside software. Currently, RetroPath is used through KNIME for which the installation and usage can be challenging. All of these features enable RetSynth to perform more comprehensive and system-wide metabolic studies than is currently available from other tools.

### RetSynth graphical user interface mode

**Fig. 7:**
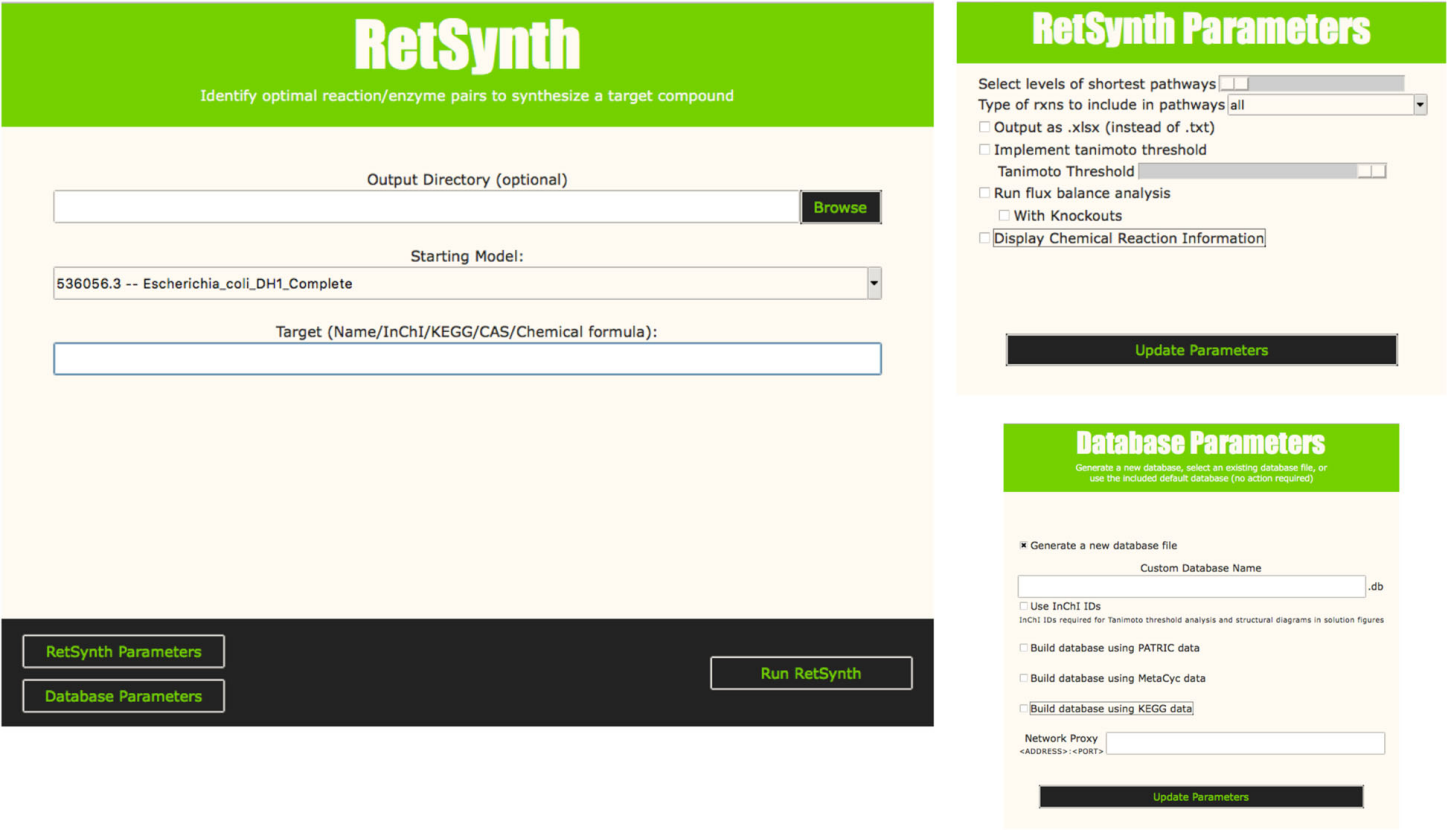
RetSynth Application. A graphical user interface for RetSynth

In addition to RetSynth’s command-line interface, a simple graphical user interface (GUI) is available for both MacOS and Windows (Fig. 7). The GUI, which was constructed with the python package Tkinter, provides the same options to the user as the command-line interface including designating a target compound and chassis organism, selecting the level of sub-optimal pathways to identify, predicting maximum theoretical yield using FBA, and the ability to generate a new custom database from metabolic repositories PATRIC, MetaCyc and/or KEGG. To save the user time, a basic default database is included with the application, allowing users to identify pathways in *Escherichia coli*. The application outputs all pathway information into figures and text/excel files to the user’s desktop or a user-specified directory. The GUI enables RetSynth to be used by a broader user-base compared to other tools currently available.

## Conclusions

RetSynth is an open-source, stand-alone software tool for identifying optimal and sub-optimal pathways to biological, chemical and hybrid production of target chemicals. Additionally, RetSynth is able to rank pathways based on maximum theoretical yield which is calculated by flux balance analysis. Our tool exceeds the capabilities of any other current software available because it includes a graphical user interface, providing the ability for RetSynth to be used by scientists without a programming background, the capability to add new and proprietary biological reactions as well as synthetic chemical databases, efficient identification of optimal and sub-optimal pathways and clear images of pathways via our visualization module to allow quick interpretation of results.

## Availability and requirements

**Project name**: RetSynth

**Project home page**: https://github.com/sandialabs/RetSynthhttps://github.com/sandialabs/RetSynth

**Operating system(s)**: Mac, Windows and Linux

**Programming language**: Python and Java

**Other requirements**: GNU Linear Programming Kit (v4.64), libSMBL

**License**: BSD 2-clause license

## Additional file

Additional file 1 Supplementary Methods-Preventing Cyclic pathways from being identified as viable routes. Outlines how software prevents pathways with cycles from being identified. (DOCX 3 kb)

## Data Availability

All software and data are available at https://github.com/sandialabs/RetSynth.

## Abbreviations

EMP: Enzyme and metabolic pathways
FBA: Flux balance analysis
GUI: Graphical User Interface
KEGG: Kyoto encyclopedia of genomes and genes
MILP: Mixed integer linear program
MINE: Metabolic in-silico network expansion
UM-BBD: University of Minnesota Biocatalyst/Biodegradation database
