# Supplementary material for: RetSynth: determining all optimal and sub-optimal synthetic pathways that facilitate synthesis of target compounds in chassis organisms
Source: BMC Bioinformatics. 2019 Sep 9;20:461. doi: 10.1186/s12859-019-3025-9 (PMC6734243; doi:10.1186/s12859-019-3025-9)
Supplement: Supplementary file 1 — Supplementary Methods-Preventing Cyclic pathways from being identified as viable routes. Outlines how software prevents pathways with cycles from being identified. (DOCX 3 kb) [file 12859_2019_3025_MOESM1_ESM.docx]

**Supplementary Methods: Preventing Cyclic pathways from being identified as viable routes:**

As each optimal pathway is identified it is checked for the presence of infeasible cycles. To identify a cycle the number of nodes (compounds and reactions) in a pathway is counted as is the number of edges (connections between nodes) and if the number of edges are greater than the number of nodes minus one a cycle is present in the pathway [1]. If a cycle exists constraints are then implemented in the same manner as was described in equation (5) in the main paper to prevent this pathway from being identified and then software runs the algorithm again to find a functional pathway.

1. Planes, F.J. and J.E. Beasley, *A critical examination of stoichiometric and path-finding approaches to metabolic pathways.* Brief Bioinform, 2008. **9**(5): p. 422-36.
